# Flawed analysis and unconvincing interpretation: a comment on Chapron and Treves 2016

**DOI:** 10.1098/rspb.2017.0273

**Published:** 2017-11-22

**Authors:** Erik R. Olson, Shawn M. Crimmins, Dean E. Beyer, Daniel R. MacNulty, Brent R. Patterson, Brent A. Rudolph, Adrian P. Wydeven, Timothy R. Van Deelen

**Affiliations:** 1 Natural Resources, Sigurd Olson Environmental Institute, Northland College, Ashland, WI, USA; 2 Timber Wolf Alliance, Sigurd Olson Environmental Institute, Northland College, Ashland, WI, USA; 3 Department of Forest and Wildlife Ecology, University of Wisconsin—Madison, Madison, WI, USA; 4 Wildlife Division, Michigan Department of Natural Resources, Marquette, MI, USA; 5 Department of Wildland Resources, Utah State University, Logan, UT, USA; 6 Ontario Ministry of Natural Resources and Forestry, Wildlife Research and Monitoring Section, Peterborough, Ontario, Canada K9 J 7B8; 7 Wildlife Division, Michigan Department of Natural Resources, Lansing, MI, USA

Chapron & Treves [1] (hereafter C&T) believe that quantifying poaching is ‘one of the most crucial questions for the conservation of large carnivores’ (p. 2). We agree that evaluating poaching is important and merits rigorous attention. Yet, we argue that C&T's claim, ‘allowing culling increases poaching’, is not supported by their data. We assert that C&T is based on flawed analysis and unconvincing interpretation of scientific literature.

C&T claimed to ‘present the first quantitative evaluation of the hypothesis that culling will reduce poaching’. However, Olson *et al*. [2] used empirical data (fates of wolves) to demonstrate that illegal killing decreases with increasing availability of lethal depredation management (hereafter, LDM). C&T claimed to ‘show that allowing wolf [*Canis lupus*] culling was substantially more likely to increase poaching than to reduce it’. However, C&T produced no empirical evidence of increased poaching, but only showed a marginal association between policy change allowing LDM and reduction in expected wolf population growth in Wisconsin and Michigan (USA). Additionally, C&T later reported a misalignment in their dataset between wolf population size, number of wolves culled and policy change [3]. C&T claim that the conclusion of their ‘paper is still supported by the correct results’ (p. 1) [3]. However, the lack of a significant change in results following the correction of their data suggests either important design flaws or a phenomena largely uncoupled from their putative ‘policy signals’.

C&T also claimed ‘replicated quasi-experimental’ (p. 2) design because changes in policy led to variation in LDM authority [1]. C&T compared ‘treatment’ periods (periods with LDM) with ‘control’ periods (when wolves were federally protected). C&T's replication claim implies independence among treatments with respect to effect of policy signals [1, p. 3], something most-certainly untrue. Authorization for LDM varied temporally [1] and spanned wolf-years [1] such that wolf-years 2003–2010 and 2012 associated with LDM authority, and 2011 and 1979–2002 did not [1, fig. 1]. Since Wisconsin and Michigan wolves increased continually during the 18 years of their analysis [4–6], the critical ‘policy signal’ was almost perfectly confounded by population size. Thus, claims of meaningful replication are dubious. C&T's analysis is a worst-case design for disentangling effects of policy and population size.

C&T selectively chose to analyse a subset of wolf population and life-history data (1995–2012), yet these datasets extend to 1980 and 1989 for Wisconsin and Michigan, respectively [5,6]. Inclusion of the full range of wolf population and life-history data would probably have produced contrary results because poaching rates were higher prior to 1985 in Wisconsin when wolves were federally protected [7]. Moreover, choosing an exponential model virtually assured that observed growth would differ from predicted growth at higher population densities because exponential growth is unsustainable due to negative density-dependence—an axiom in population biology [8]. C&T justified an exponential model by claiming that density-dependence ‘would be a weakly identifiable parameter with poaching’ (p. 5). This is an implicit admission that C&T lacked the capacity to differentiate between effects of poaching and density-dependence in their model. This suggests that C&T either misunderstood the functional link between their model structure and the biological processes emulated or they ignored this limitation.

The above concerns notwithstanding, C&T admitted that ‘negative density dependence was the most intuitive explanation for reduced growth’ (p. 5). They claimed that ‘as with prior studies on Wisconsin's wolf population’, they ‘did not detect any negative density dependence’ (p. 5). This statement is incorrect. Despite use of the plural (studies), C&T cite only Stenglein *et al*. [9] as support. However, Stenglein *et al*. [9, fig. 3] did provide evidence of negative density-dependence. C&T also ignored numerous publications demonstrating evidence of negative density-dependence in Wisconsin [4] and elsewhere (electronic supplementary material).

C&T claimed that ‘poaching was the most parsimonious explanation for observed decreases in wolf population growth rates, because [they] could rule out alternative plausible biological explanations’ (p. 5). Neither assertion is true. C&T evaluated only three life-history mechanisms for density dependence: wolf pack reproduction, wolf pack size, and area occupied by wolf packs. These three life-history features are not a comprehensive set of life-history mechanisms that could contribute to negative density-dependent growth in wolves given the scientific literature (electronic supplementary material). Additionally, while the most parsimonious hypothesis is not valid purely on the basis of simplicity, parsimonious hypotheses benefit from shorter chains of inference. Longer chains of inference require more evidence.

At least three non-exclusive hypotheses might explain the recent growth discrepancy observed by C&T: C&T's devaluation hypothesis [1], the frustration hypothesis [2] or negative density dependence (electronic supplementary material). Of these, the chain of inference for the devaluing hypothesis is longest and indistinguishable from the frustration hypothesis, while density dependence is the most parsimonious and well-supported (electronic supplementary material). C&T dismiss the frustration hypothesis [2], arguing that frustration with wolf management was present prior to LDM authority (p. 5). But there is no evidence that devaluation of wolves was exclusive to the period after implementation of LDM. Similarly, poaching has always been a feature of the population biology of Great Lakes wolves and occurred at higher rates prior to 1985 when wolves were fully protected and there was no ‘policy signal’ [7]. Frustration with wolf management probably correlates with devaluing of wolves despite C&T's assertion that devaluing depends on a specific and discrete outcome of changing policy.

C&T claimed that changes in policy associated with LDM prompted immediate devaluing of wolves, leading to increased poaching sufficient to reduce population growth. Embedded in their claim of a ‘quasi-experimental design’ [1] is an assumed cycle of public valuing and devaluing that occurred repeatedly as authority for LDM varied over time. Effectively, C&T claimed that when states had LDM authority, the public devalued wolves, and when this authority lapsed, public devaluing of wolves reversed. Such volatile and responsive shifts in values would be an unprecedented scientific finding. Behaviours of individuals change quickly [10], but aggregate behavioural change sufficient to suppress wolf population growth would require highly synchronized changes in attitudes and norms relevant not only to outcomes of this behaviour (i.e. shared desirability of removal of wolves), but also to the act of engaging in illegal activity. Further, synchronized increase in poaching sufficient to suppress wolf population growth would require not only changing behavioural intent, but would also depend upon enough individuals encountering and acting upon opportunities to poach wolves broadly across both states. Research regarding Wisconsin citizens with wolf conflicts indicated that inclinations to poach wolves were highly contextual [11]. Therefore, even among those most motivated to poach wolves, not all feasible opportunities would be acted upon equally. Furthermore, Browne-Nunez *et al*. [11] found that intentions to poach wolves remained essentially unchanged during their study period (approx. 1 yr), which coincided with wolf delisting (i.e. LDM) and part of the 2012 Wisconsin wolf harvest. Thus, C&T's assertion that liberalized culling leads to immediate devaluation of wolves and greater inclination to poach was not demonstrated [11]. Inference without adequate evidence of these behavioural dynamics is implausible.

C&T argued their results were applicable to public recreational hunting of wolves. Yet C&T only examined periods with agency culling (i.e. LDM) and did not present any analysis on potential effects of public hunting. In addition, they did not provide evidence that public attitudes of hunting and agency culling were similar. To the contrary, surveys done in both states have demonstrated that public acceptance of these types of killing is context-dependent and variable [12,13].

Growth of Wisconsin's and Michigan's wolf population has been decelerating in recent years and increased poaching probably is contributing [4,9]. C&T make strong assertions about mechanisms behind reduced growth based on a simple association with changes in policy and a biologically unrealistic model of expected growth. Inference from this association is far too weak to explain changes in illegal behaviours, which result from complex interactions of instrumental and normative influences. Yet C&T convey a level of certainty in the tone of their paper (e.g. the title, ‘… allowing culling increases poaching …’) that exceeds the quality of evidence and inference presented. C&T's analysis fails to meet even Treves's own gold or silver standards for scientific research on effectiveness of LDM [14].

## Supplementary Material

Supplemental Material

## Data accessibility

This article has no additional data.
