# Supplementary material for: Flawed analysis and unconvincing interpretation: a comment on Chapron and Treves 2016
Source: Proc Biol Sci. 2017 Nov 22;284(1867):20170273. doi: 10.1098/rspb.2017.0273 (PMC5719163; doi:10.1098/rspb.2017.0273)
Supplement: Supplemental Material [file rspb20170273supp1.docx]

**OLSON ET AL., FLAWED ANALYSIS AND UNCONVINCING INTERPRETATION: A COMMENT ON CHAPRON & TREVES 2016 - SUPPLEMENTAL MATERIAL**

**Evidence of negative density-dependence in North American wolves**

Chapron and Treves (2016, hereafter C&T) tested for density-dependence using three life history parameters, and not at the more meaningful level of population growth rate, where additive and compensatory factors reconcile (Sibly 2003). Van Deelen (2009) compared the fit of exponential growth to a suite of six density-dependent models and found no support for exponential growth in Wisconsin and Michigan wolves through 2007. Recent analyses reaffirm these density-dependent patterns using data through wolf-year 2012 (Olson, *unpubl. data*). Van Deelen (2009) also observed that density-dependence coincided with regional saturation of high-quality habitat (Mladenoff et al. 2009; Olson, *unpubl. data*) and hypothesized that dispersal in sub-optimal habitat was a mechanism - a phenomenon Treves invoked in the context of dispersal patterns within the same book (Treves et al. 2009). The dispersal hypothesis was consistent with spatiotemporal patterns of mortality in a rigorous analysis of mortality estimated from radio collared wolves (Stenglein 2014) and was demonstrated plausible for Wisconsin wolves through simulations of a spatially-explicit individual-based model (Stenglein et al. 2015a; Stenglein and Van Deelen 2016). Negative density-dependence has also been observed for Yellowstone wolves, however, intraspecific strife was the likely mechanism (Stahler et al. 2013; Cubaynes et al. 2014; cf. Mech and Barber-Meyer). Negative density-dependence has been widely described for North American wolves using a variety of analytical techniques (Table S1).

**Table S1**. Papers reporting negative density-dependence in population growth or a component of population growth for wolves (*Canis lupus*) in North America

| Citation | Location | Nature of evidence | Mechanism^a^ |
| --- | --- | --- | --- |
| Hayes and Harestad 2000 | Yukon, Canada | Yearly trends in growth rates and density | Competition for prey |
| Post et al. 2002 | Isle Royale National Park, Michigan, USA | Growth model fit to annual population estimates | Not identified |
| Patterson and Murray 2008 | Algonquin Provincial Park, Ontario, Canada | Growth model fit to annual population estimates, Regression of r_t_ on N_t_^b^ | Not identified |
| Van Deelen 2009 | Wisconsin and Michigan, USA | Growth Models fit annual population estimates | Not identified |
| Cariappa et al. 2011; (see also McRoberts & Mech 2014; Mech & Barber-Meyer 2015) | 32 sites in North America | Statistical support for type 2 and 3 numerical responses in wolf density on prey biomass regressions | Intraspecific strife, territoriality, or something other than prey limitation |
| Stahler et al. 2013 | Yellowstone National Park, Wyoming, USA | Regression analyses of demographic parameters on annual population estimates. | Intraspecific competition leading to reduced litter sizes and pup survival |
| Cubaynes et al. 2014 | Yellowstone National Park, Wyoming USA | Capture-Recapture models fit to telemetry data | Social aggression leading to reduced adult survival at high density |
| Mech and Fieberg 2014 | Isle Royale National Park (Michigan), Denali National Park (Alaska), Minnesota; USA | Growth Models fit annual population estimates | Not identified |
| Stenglein et al 2015a | Wisconsin, USA | Individual-based model parameterized with empirical data | Reduced mate-finding among dispersing wolves |
| Stenglein et al. 2015b | Wisconsin, USA | Integrated population model using annual population estimates & radio-telemetry data | Not identified |
| Stenglein and Van Deelen 2016 | Wisconsin, USA | Individual-based model, Bayesian Penalized Splines | Dispersal interacting with Spatially structured mortality |

^a^ Demonstrated or inferred; ^b^r_t =_ ln(N_t+1_/N_t_); N = population estimate, t indexes time (years)

**Evaluation of life history mechanisms and negative density-dependence**

C&T claimed that “…poaching was the most parsimonious explanation for observed decreases in wolf population growth rates, because [they] could rule out alternative plausible biological explanations” (p.5). However, C&T evaluated only three life history features as potential component mechanisms for density-dependence: wolf pack reproduction, wolf pack size, and area occupied by wolf packs.

C&T estimated support for negative density-dependence using Bayesian techniques to evaluate slopes for linear relationships between each component and density. Imposing a rigid functional form of density-dependence (linear) precludes identification of more complex functional forms of negative density-dependence (Williams 2012; Stenglein and Van Deelen 2016). For example, area occupied by wolves in Wisconsin does demonstrate negative density-dependence (Stenglein and Van Deelen 2016; Olson, *unpubl. data*). Moreover, C&T’s assumption that total area occupied is a sufficient representation of density-related effects on wolf territory size is not supported in the literature (Rich et al. 2012). Regardless, C&T may have failed to detect evidence of density-dependence for this variable because they only examined data from 2000 to 2011, when data are available as far back as 1980 for Wisconsin.

Also, C&T crudely estimated reproduction as a binary outcome (packs reproduce or not, yearly) and thereby failed to capture critical variation in litter size or pup survival (Brainerd et al. 2008; Stahler et al. 2013). C&T made *post hoc* estimates of reproductive performance using existing data (i.e., indirect estimates of changes in wolf pack size and presence of pups) (Chapron and Treves 2016). Such estimates would result in binary outcomes where pack reproduction is classified as *likely reproduced* or *undetected*, inadequate classifications for assessing density-dependence.

Additionally, these life history features are not a comprehensive set of alternate mechanisms that could contribute to negative density-dependent growth in wolves. Comprehensive mechanistic understanding of population growth may be impossible for species with complex life-histories living in variable environments; hence population biologists rely on growth as an integrator of interacting biotic and abiotic factors that influence vital rates (Sibly and Hone 2003). A demographic response to density (i.e. one observable in terms of population growth) is evidence of at least one instance of density-dependence in a component life history mechanism that influences growth (Berec et al. 2007). These components may not be identifiable with routine monitoring data and may interact in an additive or compensatory fashion (Berec et al. 2007). C&T’s cursory examination of three potential components of growth cannot “eliminate alternative plausible biological interpretations.” Thus, C&T fail to adequately account for negative density-dependence, which has been clearly documented for North American wolf populations, including those in Wisconsin and Michigan (Table 1).

**Interpretation of social science literature**

To support their inferences C&T challenge the frustration hypothesis (Olson et al. 2015a; Chapron and Treves 2016) stating, “Studies…have repeatedly shown that liberalized wolf killing did not reduce inclination to poach… [43, 44],” and “intentions [to poach wolves] rose in parallel with liberalized culling [44]…” (C&T’s citation 43 and 44 refer to Browne-Nunez et al. 2015 and Treves et al. 2013). Treves et al. (2013) did find that inclination to illegally kill wolves increased from 2001 or 2003 to 2009, a period that coincidentally overlapped with highly inconsistent LDM authority (Chapron and Treves 2016, table s1). However, C&T’s framing suggests the entire period was a period of liberalized culling, when in fact LDM was highly inconsistent (e.g., wolf status changed twice in 2009, Treves et al.’s [2013] comparison year). Browne-Nunez et al. (2015) demonstrated that local people were frustrated with inconsistency in management and felt a lack of empowerment in dealing with wolves linked to the loss of LDM authority. Highly erratic LDM would likely influence attitudes or behavioral intentions differently than consistent LDM (Olson et al. 2015a).

C&T also state, “…and the penalties for wolf poaching did not change” (p.5) and cite Refsnider (2009) for support. This is misleading as well. Penalties for illegal killing of wolves changed with federal and state status of wolves. When wolves are listed as a federally endangered species the penalties are very substantial (Refsnider 2009), but decline drastically under state listing (Wisconsin DNR 1999). Furthermore, Refsnider (2009) makes no such statement. Enforcement against all illegal take of wolves has continued regardless of their legal status.

**Parsimony**

C&T argue that “…poaching was the most *parsimonious* explanation for observed decrease in wolf population growth rates…” (Chapron and Treves 2016, p.5, emphasis added) because of a “policy signal” generated by states’ authority to conduct lethal depredation management (LDM). While a parsimonious hypothesis is not more valid simply on the basis of simplicity (truth can be complicated), parsimonious hypotheses benefit from shorter chains of inference. Longer chains of inference require more evidence supporting each additional link.

There are at least three non-exclusive hypotheses offered to explain recent reduced growth rates for wolves in the Southern Lake Superior Region (Figure 1). The first (density-dependence) is that negative density-dependence in vital rates caused reduction in growth as the wolf population increased. This hypothesis is supported by empirical data for Great Lakes wolves (Vucetich and Peterson 2004; Van Deelen 2009; Stenglein and Van Deelen 2016), observations on the behavior of other colonizing or recovering populations (see earlier sections on density dependence) and is firmly grounded in ecological theory (Mills 2013).

The second hypothesis (frustration) is that local frustration with on-again, off-again authority for LDM coupled with increasing wolf numbers and increasing wolf depredations (Olson et al. 2015b) drove increased frustrations among stakeholders (Browne-Nunez et al. 2015) leading to increased poaching sufficient to cause population effects. This theory too has empirical support for a critical link (increasing frustration, sense of diminished empowerment among stake holders, increased negative attitudes, increased illegal killing) (Treves et al. 2013; Browne-Nunez et al. 2015; Olson et al. 2015a). However, the second intermediate link has not been established (figure 1): there is no evidence that increased poaching during the time series exceeded compensatory mechanisms and reduced growth. Compensation for a given source of mortality increases with population density (Kautz 1990; Péron 2013) and C&T’s finding that population growth was unrelated to the actual number of wolves killed through LDM, despite an informative Bayesian prior essentially enforcing super-additivity, is consistent with high compensation for human-caused mortality. Analysis of radio-collared wolves indicated that rates of poaching and other human-caused mortality were highly variable and generally increasing during 1995-2012 while rates of natural mortality generally decreased in a manner consistent with compensation (Stenglein 2014). A plausible variation of the frustration hypothesis is that human-caused mortality (poaching and non-poaching) may itself be a density-dependent component of growth and this mechanism was clearly operating prior to C&T’s putative policy signal (Olson et al. 2015a).

The third hypothesis (devaluing, Chapron and Treves 2016) asserts that initiation of LDM, apart from its actual implementation, devalued wolves sufficiently among would-be poachers such that poaching increased enough to exert population effects. This chain has an additional link unsupported by empirical evidence. There are no measures of changes in valuation of wolves in the Great Lakes region concurrent with policy changes. C&T suggested that implementing LDM produced a “policy signal” or “a negative message about the value of wolves or that poaching prohibitions would not be enforced” (p. 5). Yet, even intentional, clearly communicated policy changes rarely produce such immediate and clear behavioral responses among stakeholders (Triezenberg et al. 2016). It is reasonable to assume a “policy signal” could result in a misguided public interpretation of lax enforcement policy. It is not reasonable to assume subsequent changes in poaching behavior would be strong enough and fast enough to suppress population growth when clearly targeted communications campaigns encouraging legal harvest under liberal regulations fail to produce even modest increases in harvest (Triezenberg et al. 2016). C&T proposed that psychological theory of hazard assessment may explain how poaching behavior was influenced, and C&T cite a planned communication experiment in which failure to communicate information about and benefits of bears led to diminished public acceptance (Slagle et al. 2013). C&T provide no explanation for why direct and intentional communications campaigns regarding benefits of bears produced only a small change in attitudes towards bears (Slagle et al. 2013), and yet, indirect, unintentional, and informal “signals” of implied changes in enforcement policy or diminished value of wolves would produce such a strong behavioral response (i.e., poaching with population effects).

Of these three hypotheses, the chain of inference is shortest for the density dependence hypothesis, which also accommodates the frustration hypothesis if one allows that frustration-driven human-caused mortality (Olson et al. 2015a) can be a component mechanism. The chain of inference for the devaluing hypothesis (Chapron and Treves 2016) is longest and indistinguishable from the frustration hypothesis without convincing evaluation of key intermediate links. C&T dismiss the frustration hypothesis, arguing that frustration with wolf management was present prior to LDM authority (p. 5). But there is no evidence that devaluation of wolves was exclusive to the period after implementation of LDM. Similarly, poaching has always been a feature of the population biology of Great Lakes wolves and occurred at higher rates prior to 1985 when wolves were fully protected and there was no “policy signal” (Stenglein 2014). In truth, general frustration with wolf management probably correlates with devaluing of wolves despite C&T’s assertion that devaluing depends on a specific and discrete outcome of changing policy. Hence it makes no sense to justify the devaluing hypothesis on the basis of parsimony.


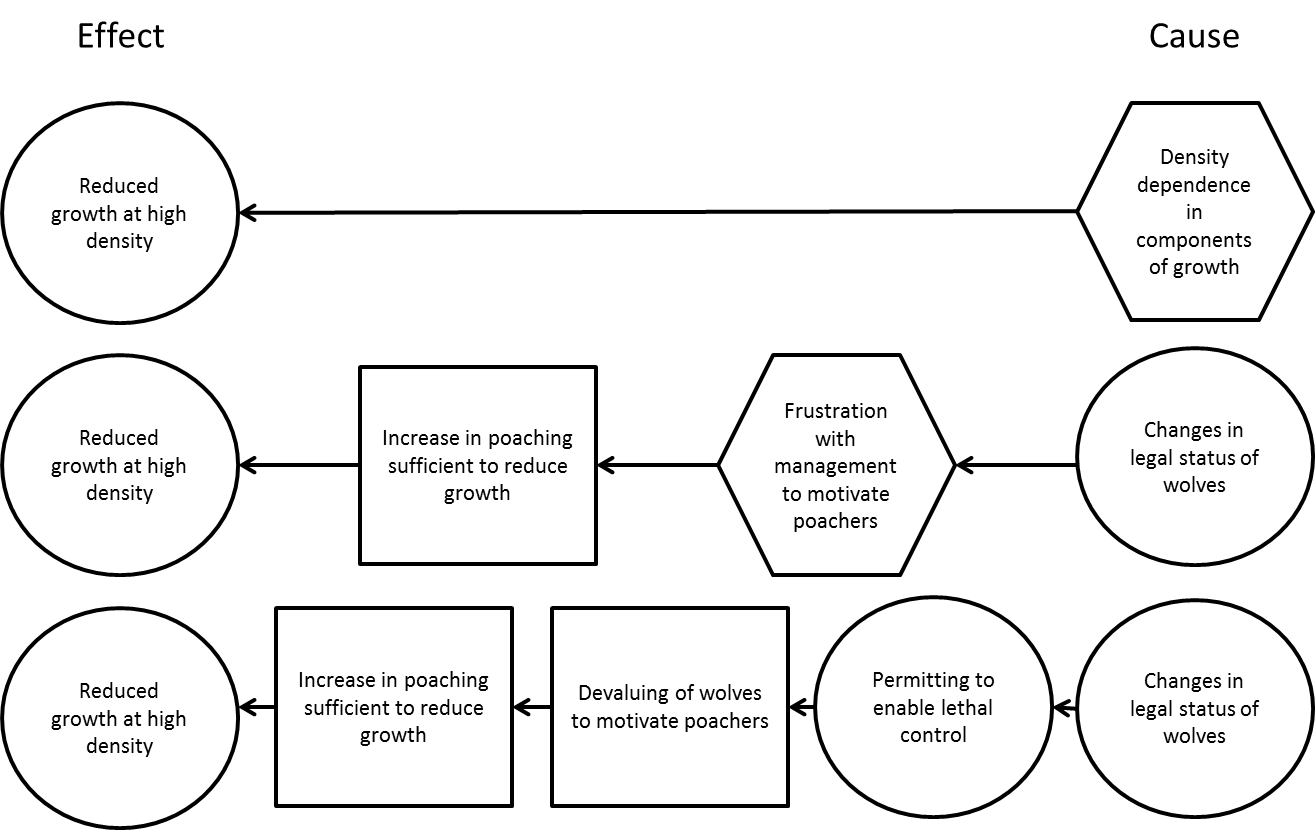


**Figure 1.** Schematic representation of chains of inference needed to support 3 hypotheses to explain observations of reduced population growth in Great Lakes wolves 1995-2012. Hypotheses are 1) Density dependence (top), Frustration (middle) and Devaluation (bottom, see text for a fuller explanation). Links that are fully observed are represented as circles. Links that have been demonstrated from empirical data and have strong theoretical support are represented as hexagons. Links that have not been demonstrated from empirical analysis or have limited theoretical support are illustrated as rectangles.

**References**

Berec L, Angulo E, Courchamp F. 2007. Multiple Allee effects and population management. Trends in Ecology & Evolution. 22(4):185–91.

Brainerd SM, Andren H, Bangs EE, Bradley EH, Fontaine JA, Hall W, Iliopoulos Y, Jimenez MD, Jozwiak EA, Liberg O, Mack CM, Meier TJ, Niemeyer CC, Pederson HC, Sand H, Schultz RN, Smith DW, Wabakken P, Wydeven AP. 2008. The effects of breeder loss on wolves. Journal of Wildlife Management 72(1):89-98.

Browne-Nunez C, Treves A, MacFarland D, Voyles Z, Tung C. 2015. Tolerance of wolves in Wisconsin: A mixed-methods examination of policy effects on attitudes and behavioral inclinations. Biological Conservation 189:59-71.

Cariappa CA, Oakleaf JK, Ballard WB, Breck SW. 2011. A reappraisal of evidence for regulation of wolf populations. Journal of Wildlife Management 75:726-730.

Chapron G, Treves A. 2016. Blood does not buy goodwill: allowing culling increases poaching of a large carnivore. The Royal Society B 283: <http://dx.doi.org/10.1098/rsp.2015.2939>

Cubaynes S, MacNulty DR, Strahler DR, Quimby KA, Smith DW, Coulson T. 2014. Density-dependent intraspecific aggression regulates survival in northern Yellowstone wolves (*Canis lupus*). Journal of Animal Ecology 83:1344-1356.

Hayes RD, Harestad AS. 2000. Demography of a recovering wolf population in the Yukon. Canadian Journal of Zoology 78:36-48.

Kautz JE. 1990. Testing for compensatory responses to removals from wildlife populations. Transactions of the North American Wildlife and Natural Resouces Conference 55:527-533.

McRoberts RE, Mech LD. 2014. Wolf population regulation revisited – again. Journal of Wildlife Management 78:963-967.

Mech LD, Barber-Meyer S. 2015. Yellowstone wolf (*Canis lupus*) density predicted by elk (*Cervus elaphus*) biomass. Can. J. Zool. 93:499-502.

Mech LD, Fieberg J. 2014. Growth rates and variances of unexploited wolf populations in dynamic equilibria. Wildlife Society Bulletin 39:41-48.

Mills LS. 2013. Conservation of wildlife populations: demography, genetics, and management, second edition. Wiley-Blackwell, West Sussex, UK.

Mladenoff DJ, Clayton MK, Pratt SD, Sickley TA, Wydeven AP. 2009. Change in occupied wolf habitat in the northern Great Lakes region. Pp. 119-138 in Wydeven

Olson ER, Stenglein JL, Shelley V, Rissman AR, Browne-Nunez C, Voyles Z, Wydeven AP, Van Deleen TR. 2015a. Pendulum swings in wolf management led to conflict, illegal kills, and legislated wolf hunt. Conservation Letters. 8:351-360.

Olson ER, Van Deelen TR, Wydeven AP, Ventura SJ, MacFarland DM. 2015b. Characterizing wolf-human conflicts in Wisconsin, USA. Wildlife Society Bulletin 39:676-688.

Patterson BR, Murray DL. 2008. Flawed population viability analysis can result in misleading population assessment: a case study for wolves in Algonquin park, Canada. Biological Conservation 141:669-680.

Péron G. 2013. Compensation and addititvity of anthropogenic mortality: life-history effects and review of methods. Journal of Animal Ecology 82:408-417 (doi:10.1111/1365-2656.12014).

Post EN, Stenseth C, Peterson RO, Vucetich JA, Ellis AM. 2002. Phase dependence and population cycles in a large-mammal predator-prey system. Ecology 83:2997-3002.

Refsnider RL. 2009. The role of the Endangered Species Act in Midwest wolf recovery. Pages 311-329 in Wydeven AP, Van Deelen TR, Heske EJ (editors). Recovery of gray wolves in the Great Lakes Region of the United States: an endangered species success story.

Rich, LN, Mitchell MS, Gude JA, Sime CA. 2012. Anthropogenic mortality, intraspecific competition, and prey availability influence territory sizes of wolves in Montana. Journal of Mammalogy 93(3):722-731.

Sibly RM, Hone J. 2003. Population growth rate and its determinants: an overview. Pages 11-40 in Sibly RM, Hone J, Clutton-Brock TH (editors) Wildlife population growth rates. Cambridge University Press, Cambridge UK.

Slagle K, Zajac R, Bruskotter J, Wilson R, Prange S. 2013. Building tolerance for bears: a communications experiment. Journal of Wildlife Management 77:863-869. (doi: 10.1002/jwmg.515).

Stahler DR, MacNulty DR, Wayne RK, vonHoldt B, Smith DW. 2013. The adaptive value of morphological, behavioral and life-history traits in reproductive female wolve. Journal of Animal Ecology 82:222-234.

Stenglein JL. 2014. Survival of Wisconsin’s gray wolves from endangered to harvested, 1980-2013. Dissertation, University of Wisconsin-Madison, USA. 172pp.

Stenglein JL, Gilbert JH, Wydeven AP, Van Deelen TR. 2015a. An individual-based model for southern Lake Superior wolves: a tool to explore the effect of human-caused mortality on a landscape of risk. Ecological Modeling 302:13-24 (<http://authors.elsevier.com/a/1Qa9F15DJ~gxjR>).

Stenglein JL, Zhu J, Clayton MK, Van Deelen TR. 2015b. Are the numbers adding up? Exploiting discrepancies among complementary population models. Ecology and Evolution 5:368-376.

Stenglein JL, Van Deelen TR. 2016. Demographic and component Allee effects in southern Lake Superior gray wolves. PLoS ONE 11(3): e0150535. doi:10.1371/journal.pone.0150535.

Treves A, Martin KA, Wiedenhoeft JE, Wydeven AP. 2009. Dispersal of gray wolves in the Great Lakes region. Pages 191-204 in Wydeven AP, Van Deelen TR, Heske EJ (editors). Recovery of gray wolves in the Great Lakes Region of the United States: an endangered species success story.

Treves A, Naughton-Treves L, Shelley V. 2013. Longitudinal analysis of attitudes toward wolves. Conservation Biology 27:315-323.

Triezenberg HA, Riley SJ, Gore ML. 2016. A test of communication in changing harvest behaviors of deer hunters. Journal of Wildlife Management 80:941-946. (doi: 10.1002/jwmg.21078).

Van Deelen TR. 2009. Growth characteristics of a recovering wolf population in the Great Lakes Region. Pages 139-153 in Wydeven AP, Van Deelen TR, Heske EJ (editors). Recovery of gray wolves in the Great Lakes Region of the United States: an endangered species success story.

Vucetich JA, Peterson RO. 2004. The influence of prey consumption and demographic stochasticity on population growth rate of Isle Royale wolves Canis lupus. Oikos 107:309-320.

Williams CK. 2012. Accounting for wildlife life-history strategies when modeling stochastic density-dependent populations: a review. The Journal of Wildlife Management 77:4-11.

Wisconsin DNR. 1999. Wisconsin Timber Wolf Recovery Plan. Wisconsin Endangered Resources Report 50. Wisconsin Department of Natural Resources, Madison, Wisconsin, USA.
